# The value of consumer neuroscience research for contemporary marketing knowledge

**DOI:** 10.3389/fnhum.2023.1214848

**Published:** 2023-06-16

**Authors:** Katrin Haidinger, Monika Koller

**Affiliations:** Department of Marketing, Vienna University of Economics and Business, Vienna, Austria

**Keywords:** consumer neuroscience, consumer research methods, marketing, big data, neuroscientific methods

## Introduction

Although consumer neuroscience offers great potential, research in the field is still scarce, particularly when compared to the application of other empirical methods. In this opinion article, we want to reflect upon the potential additional value of consumer neuroscience in selected areas of marketing, while also referencing other more recent approaches such as big data. We base our elaboration on qualitative insights gained from an exploratory look at consumer neuroscience papers with regard to the additional value for marketing. This will provide the basis for suggestions for future research in this field.

One reason why the number of consumer neuroscience papers in marketing is still lower than other empirical papers could lie in the potential uncertainty among researchers about whether consumer neuroscience can actually provide insights that are of significant relevance for marketing academics and practitioners, beyond conventional research methods (Plassmann and Karmarkar, 2015; Lee et al., 2018). Moreover, resource-intensity as well as methodological and ethical issues mentioned in the literature may be another potential explanation for the relatively slow development of consumer neuroscience (Javor et al., 2013). Consumer neuroscience papers have been published in multiple disciplines (Smidts et al., 2014), which may make it harder for marketers to appreciate their value if there is a lack of familiarity with journals from other disciplines.

In contrast, big data, as another alternative approach to self-reports, has already gained significant importance for applied marketing. It offers benefits for marketing over conventional research methods due to access to a high amount of real-time information in a natural environment (Erevelles et al., 2016).

The goal of this opinion article is to outline areas where consumer neuroscience can provide insights relevant for marketing academics and practitioners beyond conventional research methods. To do so, we took an exploratory look at the findings of consumer neuroscience papers, particularly in advertising, branding, and product management. By also touching briefly on big data, we are able to provide another outlook of the prospects of consumer neuroscience in marketing in conjunction with other methods.

## Evolution of consumer neuroscience

A deep understanding of consumers is undoubtedly a significant component of successful marketing. Conventional research methods based on self-reports, such as questionnaires, interviews, focus groups, or behavioral experiments, offer the advantage of high acceptance, but provide only limited insights into the subconscious processes of consumers, which play a significant role in decision-making processes (Plassmann and Karmarkar, 2015). Consumer neuroscience can generate insights into neural mechanisms, such as emotion, reward, memory, and attention, which are central to explaining consumer behavior and consumer decision making (Solnais et al., 2013; Camerer and Yoon, 2015; Wolf and Ueda, 2021). The application of neuroscientific methods can provide more objective insights into consumer preferences and decision making by eliminating socially desirable answers or strategic behavior, as well as recall and response biases (Camerer et al., 2005; Hubert and Kenning, 2008; Kenning and Plassmann, 2008; Reimann et al., 2011; Yoon et al., 2012; Balconi and Sansone, 2021; He et al., 2021). By looking at neuroscientific and psychophysiological processes, we can gain a deeper understanding of consumers and contribute to existing marketing knowledge (Venkatraman et al., 2012, 2015; Smidts et al., 2014). Hence, consumer neuroscience offers a lot of potential. To move the field forward, it is important to reflect upon the potential additional value of consumer neuroscience in more detail. There are three types of additional value, providing: (1) completely new insights that are not able to be obtained with conventional research methods, (2) complementary insights, in terms of explaining consumer behavior and the effectiveness of certain marketing actions not able to be explained with conventional methods, and (3) confirmatory insights, which means confirming knowledge that has been generated with traditional self-report methods by adding a neuroscientific or psychophysiological description.

## Areas of additional value of consumer neuroscience

Based upon our qualitative exploration, we will now outline important areas of application for which consumer neuroscience could provide valuable insights.

## Advertising stimuli and communications

The additional value of consumer neuroscience has been especially evident in the field of advertising, for example, when testing the effectiveness of different components of advertisements. It can help to gain a deeper understanding of the consequences of certain marketing actions, which would potentially remain unobserved when relying solely on conventional research methods. Marketers can benefit from the potential of neuroscientific methods during the creation phase of marketing activities by testing the effect of stimuli pre-launch (Rossiter et al., 2001). Plassmann et al. (2007) provide further insights into the additional benefits of applying neuroscience to gain a better understanding of how advertising works. At the same time, they discuss limitations and propose directions for further research in this area. Since then, consumer neuroscience has gained further attention in marketing research, especially through review articles on the emergence and development of the topic (e.g., Lee et al., 2018), special sessions at major conferences in marketing (e.g., at the European Academy of Marketing, Koller and Lee, 2016), and, for example, a special issue published in the Journal of Marketing Research (Camerer and Yoon, 2015).

Moreover, consumer neuroscience also supports attempts to explain the effectiveness of already implemented activities. The application of neuroscientific methods is especially valuable when the performance of certain marketing activities cannot be fully explained by conventional research methods. For instance, Guerrero Medina et al. (2021) looked at the effect of CSR messages on consumer behavior. Derived from previous literature, they argued that it was difficult for companies to translate their CSR communications into an increase in sales. By applying a neuroscientific method, they were able to identify possible reasons for that, which would have remained undetected if only traditional research techniques had been applied.

Consumer neuroscience can also be helpful when studying topics that are at high risk of being influenced by a social desirability bias. In a study by Vezich et al. (2017), consumer self-report data suggested a higher liking of green ads over controls, whereas fMRI data showed the opposite.

## Branding and product attributes

The field of branding can also benefit from neuroscientific methods. Consumer brand perception as well as brand associations are highly influenced by implicit mechanisms, which are difficult to study with conventional research methods. Regarding brand associations, in a study by Camarrone and van Hulle (2019), conventional and neuroscientific methods produced divergent results for two brands. Neuroscientific methods revealed a difference in associations between the two brands, while self-reports did not. Research on attitudes toward brands can also benefit from applying consumer neuroscience techniques (Walla et al., 2011).

Consumer neuroscience can also help when there are contradictory insights on a specific topic. The methodological limitations of conventional methods could be one reason for contradictions in the literature (Wolf and Ueda, 2021). The application of neuroscientific methods can help to provide objective clarity for these findings. For example, there has been a debate in the literature on whether brands are perceived as human-like beings or rather like cultural objects; neuroscientific studies hint at the latter (Yoon et al., 2006; Javor et al., 2018).

The application of neuroscientific methods can also be useful in the field of product evaluation as it can reveal, for example, fine distinctions in the evaluation and perception of product attributes. For instance, Frost et al. (2015) observed, in contrast to expectations, a greater activation of areas responsible for taste intensity for wines with a low alcohol level than for wines with higher alcohol levels.

## Prediction

Furthermore, neuroscientific methods have proven to be meaningful methods for testing or predicting the success of various marketing stimuli (Kühn et al., 2016), either by applying a combination of neuroscientific and conventional research methods or the application of neuroscientific methods alone. Venkatraman et al. (2015) found that the application of fMRI explains the highest level of variance of advertising elasticities, going beyond the capabilities of self-reports. Motoki et al. (2020) observed that a combination of self-report data and neuroscientific methods forecasts the viral success of advertisements on social media.

## Emotions

The neuroscientific measurement of emotions plays a significant role in predicting behavior. Pozharliev et al. (2022) observed that physiological arousal can predict consumer behavior, while self-reported affect intensity did not. Consumer neuroscience can help to shed light on conscious vs. unconscious emotions. Bettiga et al. (2020) found that consumers are aware of their emotions regarding hedonic products but not for functional products, implying that insights gained from conventional methods can be biased and incomplete. When marketers want to assess consumers' emotions about functional products, the application of neuroscientific methods is potentially more effective. Bettiga et al. (2017) found conscious and unconscious arousal were two different emotional responses that influenced attitudes toward products differently.

## Consumer neuroscience vs. big data

We also explored the prospect of consumer neuroscience by contrasting it with the potentials of big data. The two methodological approaches differ in their characteristics but can also work as a meaningful complement. For instance, in the field of creating advertisements, neuroscientific methods can support marketers, especially in testing certain marketing stimuli pre-launch, while big data, which is dependent on existing data, can primarily provide insights into the actual effectiveness post-launch. Additionally, in the field of branding, marketers could benefit from a combination of both methods. Data mining can capture a large amount of, for example, brand data, and is therefore a valid method from a company's perspective (Culotta and Cutler, 2016). However, as implicit perceptions of a brand play a fundamental role (Walla et al., 2017), classic self-reports as well as data mining could fall short. Moreover, while big data is capable of collecting a large amount of data on consumers or market trends, and subsequently building the base for developing marketing actions (Seung-Pyo et al., 2018), consumer neuroscience can be a helpful tool when emotions or subconscious phenomena play a fundamental role (Ramsoy et al., 2019).

## Conclusion and further research

The literature tells us that consumer neuroscience can be a powerful tool in the development phase of various marketing activities but also in explaining the effectiveness or ineffectiveness of already implemented activities. Also for making predictions, consumer neuroscience can provide value beyond the application of conventional research methods. Furthermore, consumer neuroscience can offer insights especially for activities where emotions are involved or social desirability biases exist. The application of neuroscientific tools can also help to bring clarity to contradictory findings. In contrast, for already well-established theories and models as well as topics where emotions play a subordinate role or where consumers can easily articulate their opinion, neuroscientific insights are more of a confirmatory character.

While big data is an emerging discipline, consumer neuroscience can also offer relevant insights for marketers that are not possible with both big data and conventional research methodologies based on self-reports, giving consumer neuroscience the potential to develop alongside these strong methodologies.

The characteristics of consumer neuroscience and big data widely differ and could rather act as a useful complement. Our opinion article is just a starting point in regards to the evaluation of the additional value of consumer neuroscience. We suggest conducting a more comprehensive analysis of papers published in, for example, the areas of advertising, branding, product management, and pricing, applying neuroscientific methods or big data approaches. Such a detailed analysis would enable the evaluation of potential overlaps and opportunities that can leverage the unique potentials of the two approaches. Another interesting area of future research could lie in evaluating the potential of consumer neuroscience to help handle the major challenges of today's society (Walla et al., 2014). Moreover, the translational aspects of consumer neuroscience, such as gender or culture (Braeutigam and Kenning, 2022) as well as the clinical perspective (Javor et al., 2023), should be considered in more detail. We hope that our opinion article motivates researchers to look more closely at the potential that consumer neuroscience can offer. Moreover, it would be great to also see more research dealing with the advantages, challenges, and ethical and societal concerns related to consumer neuroscience as well as big data, dealing with them both separately and in conjunction.

## Author contributions

KH was the leading author responsible for the conceptual outline, the exploration of literature, and the first draft of the manuscript. MK contributed to the revision and writing up of the provided draft. Both authors contributed to the article and approved the submitted version.
